# Intraoral scanner-based monitoring of tooth wear in young adults: 12-month results

**DOI:** 10.1007/s00784-021-04162-6

**Published:** 2021-09-08

**Authors:** Maximiliane Amelie Schlenz, Moritz Benedikt Schlenz, Bernd Wöstmann, Alexandra Jungert, Carolina Ganss

**Affiliations:** 1 Department of Prosthodontics, Dental Clinic of the Justus Liebig University Giessen, Schlangenzahl 14, 35392 Giessen, Germany; 2 Biometry and Population Genetics, Institute of Agronomy and Plant Breeding II, Interdisciplinary Research Center for Biosystems, Land Use and Nutrition (IFZ), Justus Liebig University Giessen, Giessen, Germany; 3 Department of Conservative and Preventive Dentistry, Dental Clinic of the Justus Liebig University Giessen, Giessen, Germany

**Keywords:** Tooth wear, Erosion, Attrition, Young adults, Intraoral scanner, Monitoring

## Abstract

**Objectives:**

To investigate tooth wear in young adults, intraoral scanning was used for digital monitoring of the mandibular first molar over 12 months. A possible influence of aetiological factors obtained by a questionnaire on tooth wear was investigated.

**Materials and methods:**

A total of 109 participants (mean age at the start of the study: 21.0 ± 2.2 years) were included in this clinical study. At baseline (T0), an intraoral scan (Trios 3, 3Shape) of the study tooth (FDI # 36 or #46) was conducted. After a mean observation period of 373 ± 19 days, a second intraoral scan (T1, *n* = 94) of the same tooth as at T0 was performed and standard tessellation language datasets were superimposed with 3D analysis software (GOM Inspect). The occlusal surface of the study tooth was divided into 7 areas (5 cusps, 2 ridges) and maximum vertical substance loss was measured between T0 and T1 (*n* = 91). Three types of tooth wear were defined: cupping (C), facet (F) and combined cupping-facet (CF). Furthermore, a questionnaire on aetiological factors, such as dietary behaviour, was filled out at T0. Data were analysed with non-parametric tests (*p* < 0.05).

**Results:**

Only one study tooth exhibited no tooth wear at T0, whereas 3 teeth showed C, 47 teeth F and 40 teeth CF. A progression of vertical substance loss for all three types was shown. Most affected were the mesiobuccal cusps (43, 38/47 µm; median, 95%CI) followed by distobuccal (36, 33/39 µm), mesiolingual (35, 26/40 µm), distolingual (34, 27/36 µm) and distal (31, 25/34 µm). On mesial and distal ridges, only F was detected with the lowest vertical substance loss of all areas (mesial ridge: 0, 0/0 µm; distal ridge: 0, 0/0 µm). An association between aetiological factors and loss values could not be shown.

**Conclusions:**

All study teeth showed clear signs of wear, and after only 1 year, further substance loss was detectable. This result is of significance for young adults.

**Clinical relevance:**

Since data of young adults regarding tooth wear are scarce, the results give a first idea of the amount of vertical loss per year and its relation to aetiological factors such as dietary behaviour. Therefore, further studies over a longer observation period are highly recommended.

**Supplementary Information:**

The online version contains supplementary material available at 10.1007/s00784-021-04162-6.

## Introduction

Tooth wear is defined as cumulative surface loss of mineralised tooth substance due to various physical or chemo-physical impacts not caused by caries, trauma or resorption [1]. It is not reversible and, to some extent, is part of the physiological aging of the natural dentition. However, it can turn into a pathological condition if the progression rate does not comply with the expected lifetime of the tooth [2].

Cross-sectional prevalence studies have revealed that tooth wear already occurs at a young age [3, 4]. The overall estimated prevalence of erosive tooth wear in 8- to 19-year-olds is 30.4% [4] and in approximately 5% of 7- to 18-year-olds, even dentine is exposed [3]. These findings indicate that tooth wear is a relevant oral health issue in young patients. However, little is known about whether, when and how these lesions progress. The few studies published to date have shown that an increase in the number and severity of lesions can be detected at a young age and within a few years [5–9], but we are still far from a deeper understanding of the dynamics of tooth wear in individual patients. Furthermore, tooth wear does not affect all areas of the dentition to the same extent. The mandibular first molars are the most common teeth to exhibit these lesions and show the most pronounced progression rates [5, 7, 8, 10, 11]. Therefore, they have been proposed as marker teeth for erosive tooth wear in young subjects [5, 10]. The reasons for more severe involvement of mandibular first molars than their antagonists are unknown.

Index systems are the established method for the assessment of the prevalence, incidence and progression of tooth wear [12]. However, in our view, these approaches have some drawbacks if they are used for monitoring wear. They are subjective and semi-quantitative, detect tooth wear only when macroscopic changes occur and require extended observation times. Furthermore, these approaches are selective, since they cover only specific forms of wear, and data collected with different index systems are difficult to compare. A more sensitive and objective approach could be using superimposed three-dimensional (3D) images obtained from intraoral scanners (IOSs) [13–15]. IOSs were used in a recent study with orthodontic study models of patients aged 11–13 [16]. The models were obtained at baseline and after two appointments within 29 months. In patients with visible progression of wear on molars according to the Basic Erosive Wear Examination index [17], scanner data showed a significantly higher volume loss than in those without visible wear [16]. However, using casts has an inherent problem of volume changes from the impression and cast materials as well as inaccuracies from air traps or plaster beads. Therefore, direct capture of 3D images from intraoral scanners can prove itself to be more reliable.

To date, wear analyses with 3D datasets have been carried out mainly after various types of dental restorations [18]. However, little is known about the wear processes in the natural dentition that are unrelated to dental interventions. Considering the current prevalence data, a deeper mechanistic understanding is urgently needed as a basis for effective concepts for early monitoring and prevention of tooth wear, particularly in adolescents and young adults (age between 18 and 25 years).

The present clinical prospective observational study assessed the wear process on the occlusal surfaces of mandibular first molars in young adults. The primary aim was to collect qualitative and quantitative data of the wear processes in the natural dentition of young subjects. The secondary aim was to use a specifically designed questionnaire to explore the impact of various aetiological factors. The secondary aim was to relate the data regarding quantitative loss to the assumed aetiological factors obtained from the questionnaires. The study explores the use of intraoral scanners as an innovative tool for the monitoring of tooth wear.

## Participants, materials and methods

### Study participants

The study was conducted at the Department of Prosthodontics and the Department of Conservative and Preventive Dentistry of Justus Liebig University Giessen (Germany). This study was approved by the local ethics committee of Justus Liebig University Giessen (ref. no. 148/18) and recorded in the German Clinical Trial Register (DRKS00021279). All investigations were performed in accordance with the ethical guidelines of the Declaration of Helsinki. Study participants were informed about the background and the procedure of the study and signed informed consent forms were obtained from all participants.

At the beginning of the study, the mean age of participants (68 females and 41 males) was 21.0 ± 2.2 years. The area of interest was the occlusal surface of the mandibular first molar (FDI #36 or #46). The inclusion criteria were patients aged 18–25 years at the beginning of the study, no dental caries and no visible plaque on the study tooth and the presence of an occluding antagonist to the study tooth with occlusal contact points. If a restoration was present in the study tooth or its antagonist, the inclusion criteria dictated that its extension should not exceed one-third of the occlusal surface. The exclusion criteria were severe disease [19], ongoing orthodontic treatment and crowns and bridges in the upper jaw.

### Clinical examination

**Fig. 1:**
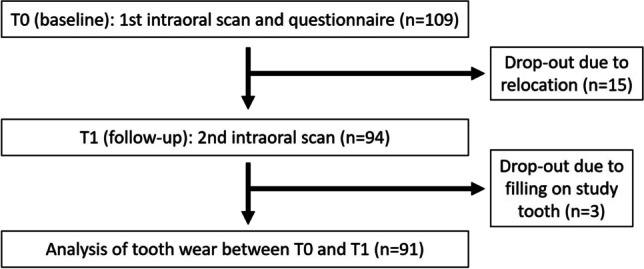
Flow scheme of the observational study

Between end of 2018 and beginning of 2020, the study teeth were investigated at baseline (T0) and follow-up (T1) with a mean observation period of 373 ± 19 days (Fig. 1). To ensure comparable testing conditions, a single operator (M.A.S.) experienced in intraoral scanning performed the clinical examination with an IOS (Trios 3, 3Shape, Copenhagen, Denmark). Before usage, the scanning tip of the IOS was calibrated using the calibration device provided by the manufacturer [20]. Dry tips (Microbrush International, Grafton, MA, USA) were used to absorb saliva from the parotid gland and the tongue was retracted with a customary wooden spatula. The teeth were gently air-dried. The same scan strategy was conducted beginning with the occlusal and oral surfaces and finishing with the buccal surface [21]. The scanning tip was guided parallel to the teeth at a constant distance of 1–1.5 cm without contact. Only the study tooth and the adjacent teeth were scanned to ensure higher accuracy of the scan dataset [22]. To ensure that artefacts such as food leftovers do not falsify the intraoral scan data, all participants brushed their teeth before the clinical examination.

After intraoral scanning, the participants were asked to fill out a questionnaire regarding several wear-related behavioural items.

### Measurement of tooth wear

**Fig. 2:**
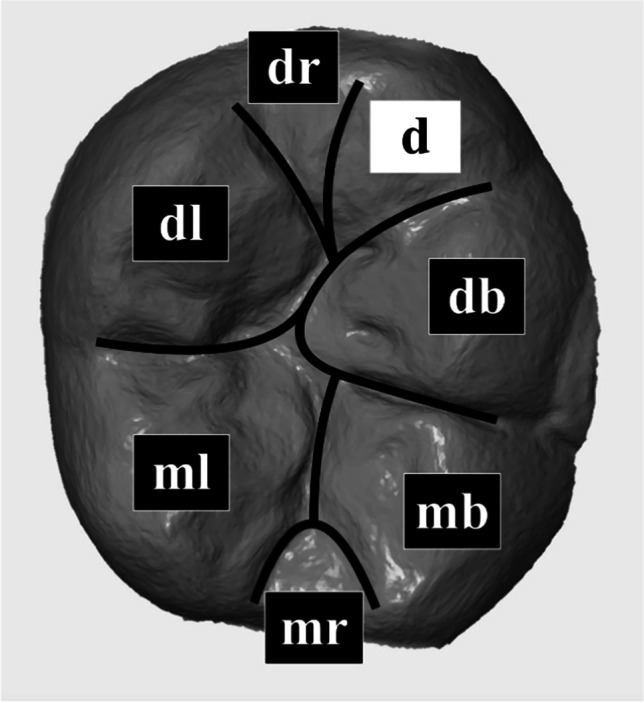
Classification of the occlusal surface of each study tooth distributed into seven areas: mesiobuccal (mb), distobuccal (db), mesiolingual (ml), distolingual (dl) and if existing distal (d) cusp as well as mesial (mr) and distal ridge (dr)

The occlusal surface of each tooth was classified into seven areas: mesiobuccal (mb), distobuccal (db), mesiolingual (ml), distolingual (dl), distal cusp (d) if present, mesial marginal ridge (mr) and distal marginal ridge (dr) (Fig. 2). Each of these areas was assessed individually.

At T0 and T1, tooth morphology was assessed using 3D images generated with the IOS. Three types of lesions were defined: cupping (C), facet (F) and combined cupping-facet (CF).

For the quantitative analysis of tissue loss, datasets at T0 and T1 were exported from the IOS software in the standard tessellation language format and imported into GOM Inspect 3D software (version V8 SR1, GOM GmbH, Braunschweig, Germany). All data points were reduced to the area of interest, which was the occlusal surface above the equator of the study tooth. For the analysis of tooth wear, datasets at T0 and T1 were superimposed using the established best-fit alignment with the iterative closest point technique for the measurement of tooth wear [15, 23]. The overall overlay error for superimposition was determined for each tooth. Superimposition of the T0 and T1 datasets revealed an error of ≤ 10 µm.

The difference in tooth wear between T0 and T1 was determined by selecting the blue-coloured surface mapping of tissue loss in the 3D analysis software and measurement of the maximum vertical tissue loss in micrometre. This procedure was performed by an experienced operator (M.B.S.).

### Questionnaire

The questions were designed in cooperation with an experienced scientific nutritionist (A.J.). Furthermore, pre-tests and expert interviews with adults aged 18–25 years were conducted before this study. In the cognitive pre-tests, the technique of thinking aloud was applied. Subsequently, the questionnaire was completed.

Wearing a nightguard and the frequency of chewing gum were considered physical influencing factors. Acid impacts were assessed using questions regarding heartburn and the frequency of consumption of various acidic food and beverages. Acid impacts per day were calculated from these frequencies. Furthermore, participants were asked how much they generally liked acidic food and beverages. The evaluated items and the equations for the calculation of the daily acid impacts are presented in the “Supplementary information” section.

### Statistical analysis

Statistical analyses were performed using IBM SPSS Statistics version 25 (IBM Germany GmbH, Ehningen, Germany).

The frequency and the type of lesions at T0 and corresponding changes at T1 were reported descriptively.

As there was a significant deviation from the Gaussian distribution (Kolmogorov–Smirnov test) for all quantitative data regarding substance loss, non-parametric test procedures were used for all statistical analyses. Values were presented as median and 95% confidence intervals obtained by bootstrapping (method of sampling: simple, number of samples: 1000). Quantitative tooth structure loss in different tooth areas was initially analysed using the Friedman test. Kruskal–Wallis test and subsequent Mann–Whitney tests were used to investigate whether the loss values differed according to the lesion type. The largest loss in terms of numbers was observed at the mesiobuccal cusp, which was also the area where the most diverse lesion types were observed. To keep the statistics straightforward, we defined this cusp as an area of particular interest and related further statistical analyses only to this region. Accordingly, loss values at the mesiobuccal cusp were compared with that at the other tooth areas (Wilcoxon test). Mann–Whitney test was used to analyse the sex differences in loss values. The association of nutrition questionnaire items and the frequency of gum chewing and wearing a nightguard was analysed using Kruskal–Wallis tests. When calculating the acid impacts, two subjects who left entire categories of questions unanswered were excluded. Single unchecked items were treated as missing values. To estimate the effect of acid impact on loss values, a correlation procedure (Spearman) was performed. The level of significance was set at *p* < 0.05.

## Results

Due to relocation, 15 participants dropped out of the study at T1 and three more participants were excluded due to restoration in the study tooth. Thus, data from 91 participants were analysed in this study.

### Morphology

At T0, 90 of the 91 teeth exhibited some type of wear. The percentage distribution of the wear types in different tooth areas is shown in Table 1. The highest number of lesions was observed at the mesiobuccal cusp (96.4% of the teeth exhibited some kind of wear), and mesial ridges showed the fewest lesions (12.1% of the teeth exhibited some kind of wear). In all regions, facets were the most common type of lesions.

**Table 1:** Distribution of lesion types [%] in the different tooth areas (mesiobuccal (mb), distobuccal (db), mesiolingual (ml), distolingual (dl) and if existing distal (d) cusp as well as mesial (mr) and distal ridge (dr) of the occlusal surface (*n.o.* not observed) for T0 and T1

Observation point	Area	Type of lesion				
		None	Cupping	Facet	Combined cupping-facet	Not present
T0	mb	6.6	16.5	56.0	20.9	
	db	15.4	1.1	74.7	8.8	
	ml	42.9	4.4	49.5	3.3	
	dl	31.9	4.4	59.3	4.4	
	d	11.0	4.4	61.5	3.3	19.8
	mr	87.9	n.o	12.1	n.o	
	dr	80.2	n.o	19.8	n.o	
T1	mb	6.6	15.4	56.0	22.0	
	db	12.1	1.1	76.9	9.9	
	ml	35.2	5.5	56.0	3.3	
	dl	30.8	4.4	60.4	4.4	
	d	9.9	4.4	62.6	3.3	19.8
	mr	83.5	n.o	16.5	n.o	
	dr	74.7	n.o	25.3	n.o	

Twenty teeth exhibited morphological changes within the observation period (Table 2). Newly developed lesions were almost exclusively facets (*n* = 17) and only one case of cupping was observed. Pre-existing facets changed to combined cupping-facet lesions in two cases.

**Table 2:** Changes in lesion type (total number of instances of change = 20) from T0 to T1 in the different areas (mesiobuccal (mb), distobuccal (db), mesiolingual (ml), distolingual (dl) and if existing distal (d) cusp as well as mesial (mr) and distal ridge (dr))

	T1 cupping	T1 facet	T1 combined cupping-facet
mb			
T0 cupping			1
db			
T0 none		3	
T0 facet			1
ml			
T0 none	1	4	
dl			
T0 none		1	
d			
T0 none		1	
mr			
T0 none		4	
dr			
T0 none		4	

### Loss values

**Fig. 3:**
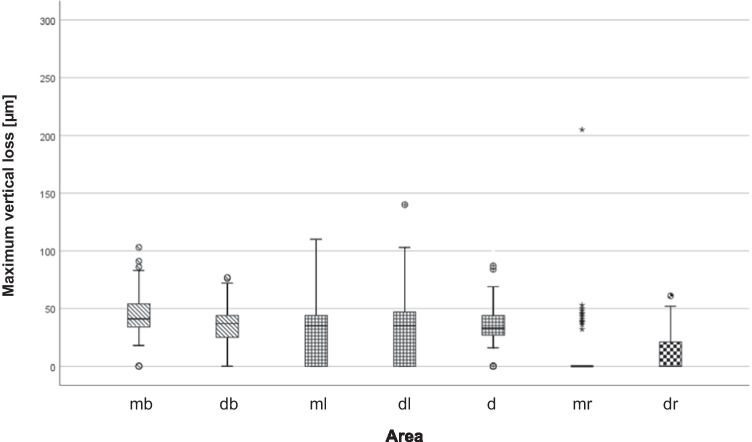
Boxplot diagram of maximum vertical loss [micrometre] for the seven areas (buccal cusps are shaded oblique, lingual cusps are checkered small and ridges are checkered big)

**Fig. 4:**
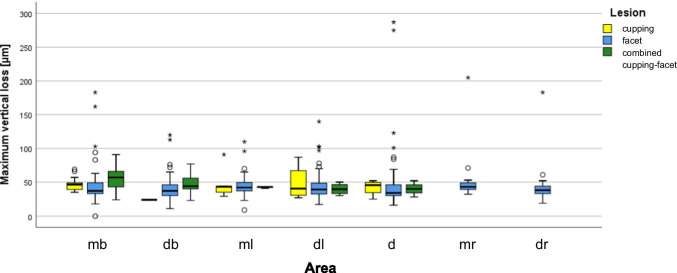
Boxplot diagram of maximum vertical loss [micrometre] for the seven areas (mesiobuccal (mb), distobuccal (db), mesiolingual (ml), distolingual (dl) and if existing distal (d) cusp as well as mesial (mr) and distal ridge (dr)) distributed to type of lesion (cupping = yellow, facet = blue, combined cupping-facet = green)

From T0 to T1, loss was found in all teeth and the distribution is shown in Figs. 3 and 4. The mesiobuccal cusp was the most affected area (43.0 [38.0–47.0] µm), followed by the distobuccal (36.0 [33.0–39.0] µm, *p* ≤ 0.001), mesiolingual (35.0 [26.0–39.97] µm, *p* ≤ 0.001), distolingual (34.0 [27.0–36.0] µm, *p* ≤ 0.001) and distal (31.0 [25.0–34.0] µm, *p* ≤ 0.05) cusps. The mesial (0 [0–0] µm, *p* ≤ 0.001) and the distal (0 [0–0] µm, *p* ≤ 0.001) marginal ridges showed the lowest loss values. The *p* values referred to comparisons of the loss values with that at the mesiobuccal cusp. At the mesiobuccal cusp, the highest loss values were found for combined lesions (57.0 [45.0–64.0] µm), followed by cuppings (47.0 [39.0–52.5] µm) and facets (37.0 [35.0–45.0] µm). The difference in loss values between facets and cuppings as well as between facets and combined lesions reached statistical significance (*p* ≤ 0.05 and *p* ≤ 0.001, respectively). The difference in loss values between cuppings and combined lesions was not significant (*p* = 0.071).

### Factors influencing loss

**Fig. 5:**
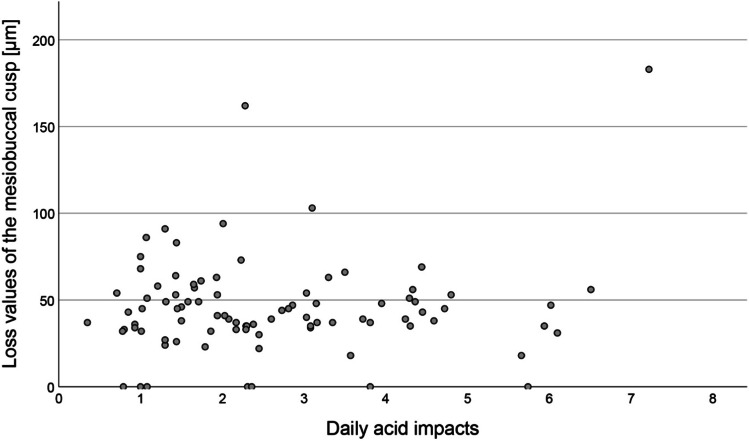
Correlation between the number of daily acid impacts and loss values [micrometre] of the mesiobuccal cusp (*n* = 89, calculation is presented in the Supplementary Information)

The results of the questionnaires and related loss values are presented in Table 3. Heartburn was reported rarely (*n* = 6); hence, it was not included in the statistical analysis. None of the questionnaire items was significantly associated with loss values. There was no significant correlation between the number of daily acid impacts and loss values (Spearman’s rho = 0.008, *p* = 0.937; *n* = 89) (Fig. 5). There was no significant difference in the loss values between men and women (*p* = 0.07).

**Table 3:** Results of the questionnaires and loss values (median and 95% confidence interval) according to the different influencing factors

Item		Category			*p* value
		Male	Female		
Sex	*n* (%)	35 (38.5)	56 (61.5)		
	Loss (µm) 95% CI	46.0 39.0;54.0	39.0 35.0;46.0		*p* = 0.070
		No	Yes		
Wearing a nightguard	*n* (%)	74 (81.3)	17 (18.7)		
	Loss (µm) 95% CI	40.5 37.0;46.5	47.0 22.0;66.0		*p* = 0.695
Consumption of chewing gum	*n* (%)	37 (40.7)	54 (59.3)		
	Loss (µm) 95% CI	41.0 37.0;49.0	43.0 38.0;49.0		*p* = 0.852
		No	Neither	Yes	
I like acidic food	*n* (%)	31 (34.1)	19 (20.9)	41 (45.1)	
	Loss (µm) 95% CI	47.0 36.0;52.0	35.0 31.0;56.0	40.0 37.0;48.0	*p* = 0.626
I like acidic drinks	*n* (%)	55 (60.4)	17 (18.7)	19 (20.9)	
	Loss (µm) 95% CI	39.0 36.0;46.5	43.0 35.0;53.0	44.0 37.0;58.0	*p* = 0.703

## Discussion

The main objective of this study was to obtain basic data on the dynamics of wear processes in natural teeth that are not related to restorations or restorative materials. We examined young adults, since wear plays a special role considering the long functional lifetime of the dentition. Intraoral scanning is being increasingly used as the method of choice for this purpose [13, 16, 24, 25], as it can measure changes in tooth morphology with sufficient accuracy [15, 24]. While other studies have used models, we used in situ scans to avoid model-related errors, thereby increasing the accuracy of measurement. In contrast to conventional methods that require an elaborate laboratory setup and expert knowledge, IOS can be easily used in every dental office. Furthermore, transfer errors of conventional impression taking and gypsum models due to the expansion and contraction of the materials can be omitted.

To obtain wear-related data, the datasets generated by the IOS must be superimposed. However, the alignment methods affect the measurement outcome [26]. In the present study, the established best-fit alignment with the iterative closest point technique was applied, which has already been described by Güth et al. [23]. Best-fit alignment may underestimate the measured tooth wear, as it minimises the mesh distance error between two datasets in contrast to the reference alignment [26]. This measurement problem arises with in vivo measurements, as there are no fixed reference areas in a natural tooth. However, we validated our method against profilometric measurements and observed sufficiently good agreement. Our methodology was also found to be suitable for substance loss in the required dimension [15]. In the present study, the superimposition error was ≤ 10 µm, which is in good agreement with the literature [23, 27].

Our research approach included both qualitative and quantitative aspects. The former consisted of recording morphologically distinct types of tooth structure loss including flat well-demarcated areas and cupped lesions, which we labelled according to their shape. We did not use the terms attrition and erosion to avoid anticipating a particular aetiology. To date, there are no generally accepted parameters for quantitative analysis. Other studies have calculated volumes, averaged the loss values from different wear areas of a tooth or examined the occlusal surface as a whole [18]. However, none of these methods was suitable for our research question, since the majority of the teeth had fissure sealants and some had fillings and losses of sealant or filling material obscured dental hard tissue losses. Therefore, the occlusal surface was segmented into areas that included the buccal and the lingual cusps as well as the marginal ridges and these areas were individually analysed. This procedure not only allowed reliable measurement of loss but also allowed differential analysis of the wear dynamics of the occlusal surface.

We examined the mandibular first molar, as this tooth is most affected by erosive tooth wear at a younger age and shows the highest incidence rate for erosive tooth wear [5, 7, 8, 10, 11]. It is not known whether this is true for other types of wear. Consistent with the findings in the literature, facets and cuppings were also very frequent in our study at T0 and only one tooth exhibited natural morphology without any alterations. The most affected area was the mesiobuccal cusp, which showed various types of wear. Interestingly, all other areas were almost exclusively affected by facets, but rarely by other lesion types. At T1, some new lesions were observed. Almost all of these developed in previously unaffected areas and almost all of them were facets. When the facets changed, they merged into combined lesions. In only one case, cupping occurred directly without the presence of a previous facet. Longer observation periods are needed to confirm whether this could be a typical developmental pathway of tooth wear.

We observed median loss values of 31–43 µm at cusps with considerable inter-individual ranges. This value seems worryingly high given the young age of our participants. However, after the comparatively short observation period, it is difficult to assess the clinical significance of this result because tooth wear is certainly not simply linear, but alternates between active phases and stagnation. Nevertheless, our study at least indicates that wear is not an issue of older age, but needs attention already in young patients. As we had expected, the mesiobuccal cusp exhibited the highest loss of substance, which was significantly higher than that observed at the other cusps. When looking at the different lesion types, cuppings and combined lesions were associated with higher loss values than facets. This indicates that higher wear rates must be expected as soon as cupping occurs. It has been suggested that such lesions are caused by accelerated physical wear of dentine, which is softer than enamel [28]. However, it is unclear whether cupped lesions expose the dentine. Rather, in vitro studies have shown that the bottom of cupped lesions can also be located in the enamel [29]. Similar results were observed in an electron microscopic study with replicas of cupped lesions in patients [10]. Therefore, the underlying mechanism responsible for this type of lesion remains unclear.

Our secondary aim was to relate loss values to aetiological factors. These include mechanical effects such as the action of antagonists in parafunction and chewing as well as chemical factors such as effects of acids from diet and stomach contents. However, there is no standardised tool to survey such factors in a generally comparable way. Therefore, we created our own questionnaire to assess the frequency of consumption of food and beverages along with basic preferences regarding nutrition. The association between wear and different types of food and beverages is inconsistent in the literature and their erosive potential can vary greatly within a category. For example, various soft drinks may have different erosive potentials. Hence, we summarised the consumption frequencies of all acidic food and beverages into acid impacts per day.

However, we could not find an association between the loss values and the frequency of acid impacts or general food and drink preferences. This finding is consistent with the results of a systematic review that reported no associations between clinical findings and dietary aspects in most cross-sectional studies [30]. Similar results were reported in two comprehensive longitudinal studies. A Dutch study [31] included 656 children aged 10–12 years. Among these, 572 were followed up after 3 years. The only risk factors identified were frequent consumption of alcoholic mixed drinks and acidic vegetables. However, the strength of the association was weak (alcoholic mixed drinks: odds ratio, 1.82; acidic vegetables: odds ratio, 1.16). A recent Brazilian study [32] included 801 12-year-olds and 680 could be followed up after two and a half years. Dietary behaviour was not a risk factor in the aforementioned study. Therefore, the significance of questionnaires in studies should be discussed and the findings interpreted with caution.

The physical factors included in the questionnaire were gum chewing and wearing a nightguard. The latter was considered to indicate parafunction such as grinding. However, similar to the nutritional parameters, no association was observed between these factors and tooth structure loss, corroborating the results of other studies [31, 33].

It is well established that acidic food and beverages as well as physical factors, in principle, can result in substance loss [34]. However, these interactions have not been shown clinically in a consistent manner to date. A possible reason is that tooth wear is generally cumulative and therefore, may not be associated with currently acting aetiological factors. Moreover, self-reported behaviour seems to reflect actual behaviour only to a limited extent [33, 35]. The adaptation of responses to social desirability plays a role, and it is also important to consider the fact that memory is not a simple reflection of past events. All these can lead to various sources of systematic error [36].

In addition to the aforementioned aetiological factors, sex could also play a role in tooth wear. Epidemiological studies have generally shown inconclusive results. However, when sex was a statistically significant factor, males consistently exhibited greater tooth wear than females [3]. Similar findings were observed in the present study. Men exhibited higher loss values than women, but the difference was not statistically significant (*p* = 0.07).

A limitation of the present study was that only the occlusal surface of a single tooth was examined in every participant. Although there are clinically relevant reasons to focus on this area, tooth wear naturally occurs in other areas of the dental arch and on surfaces other than the occlusal surface of the first molar. The accuracy of IOS in scanned areas from up to one quadrant is significantly higher than that of conventional impressions. However, transfer inaccuracies for the complete arch have been described [37, 38]. Thus, discussions are needed regarding whether the current accuracy of IOS is high enough to display tissue loss in the complete dentition at the micrometre level. After further improvements in wear measurements with intraoral scanners and better establishment of these devices, future investigations should analyse the wear dynamics in the entire dentition. Beyond that, multicentre studies should be conducted to investigate a larger number of participants and avoid bias.

Another limitation is that some potential causal factors, such as oral hygiene habits, were not included. However, as the main aim of our study was to collect wear data, the questionnaire data can only be an initial approach to deeper analytical questions. Further studies should first clarify which strategies are best suited for this purpose.

## Conclusion

The present study demonstrates that tooth wear is a relevant issue already at a young age. In the course of 12 months, the mesiobuccal cusp of the first lower molar exhibited notable loss values with a median of about 40 µm; facets, as well as cuppings and combined lesions, were found in this area. No association between loss values and the assumed causative factors was observed. The results highlight the need for innovative research strategies for the investigation of the underlying mechanism of tooth wear in young patients. The study also presents in vivo monitoring with intraoral scanners as an innovative tool for the short-term monitoring of substance loss.

## Supplementary Information

Below is the link to the electronic supplementary material.

Supplementary file1 (DOCX 21 KB)
