# Supplementary material for: Intraoral scanner-based monitoring of tooth wear in young adults: 12-month results
Source: Clin Oral Investig. 2021 Sep 8;26(2):1869–78. doi: 10.1007/s00784-021-04162-6 (PMC8816769; doi:10.1007/s00784-021-04162-6)
Supplement: Supplementary file 1 — Supplementary file1 (DOCX 21 KB) [file 784_2021_4162_MOESM1_ESM.docx]

**Questionnaire**

To investigate the number of acid impacts per day, the consumption of the following drinks and food was enquired in a questionnaire.

Drinks:

- Energy drinks
- Lemonade
- Cola/Cola mixed drinks
- Soft drinks other than cola and lemonade
- Light soft drinks
- Fruit juices
- Smoothies
- Iced tea
- Isotonic sports drinks
- Fruit tea

Food:

- Citrus fruits
- Soft fruits
- Stone fruits
- Pome fruits
- Pineapple
- Sour pickled foods
- Salad dressings with vinegar
- Sour sweets

The following answer options were available, which were considered equivalent to the acid impact per day in brackets:

- Never/rarely (0)
- 1-3x per month (0.07)
- 1-4x per week (0.36)
- 5-6x per week (0.79)
- 1-2x daily (1.5)
- 3-4x daily (3.5)
- ≥ 5x daily (5)

In addition, the consumption of wine was enquired with the following answer options available, which were considered equivalent to the acid impact per day in brackets:

- never (0)
- ≤ 1x per month (0.02)
- 2-3x per month (0.08)
- 1-3x per week (0.29)
- 4-6x per week (0.71)
- 1x daily (1)
- ≥ 2x daily (2)

The individual sums were calculated for further evaluation, thus yielding the acid impact per day.

**Example:**

Consumption frequencies of a subject:

1. Energy drinks (1-4x per week)
2. Smoothies (5-6x per week)
3. Fruit tea (1-2x per day)
4. Wine (4-6x per week)
5. Citrus fruits (1-2x daily)
6. Soft fruits (1-2x daily)
7. Salad dressings with vinegar (5-6x per week)
8. Sour sweets (1-4x per week)

Calculation (addition of corresponding acid impacts):

1. 0.36
2. 0.79
3. 1.5
4. 0.71
5. 1.5
6. 1.5
7. 0.79
8. 0.36

*Total:* ***7.51*** *acid impacts per day.*

The answers to the questions were related to the dietary behavior during the last four weeks.

The following statements should then be evaluated:

1. I like to eat sour food.

2. I like to drink sour drinks.

The following answers could be chosen:

A) I do not agree at all.

B) I rather disagree.

C) Neither.

D) I tend to agree.

E) I fully agree.

In addition, the participants were asked the following questions with the following response options:

I) Do you wear a nightguard?

a) no.

b) yes, occasionally.

c) yes, frequently.

d) yes, regularly/always.

II) Do you suffer from heartburn (regurgitation of stomach content)?

a) no.

b) yes, ≤ once a week.

c) yes, 2-3x per week.

d) yes, 4-6x per week.

e) yes, daily.

III) Do you chew gum right after you eat something?

a) no.

b) yes, occasionally.

c) yes, regularly.
